# Supplementary material for: Computationally efficient model of myocardial electromechanics for multiscale simulations
Source: PLoS One. 2021 Jul 22;16(7):e0255027. doi: 10.1371/journal.pone.0255027 (PMC8297763; doi:10.1371/journal.pone.0255027)
Supplement: S1 File — The rule of the choice of the solution of Eq (19) for Ca2+ concentration in subspace, the 0D mechanical problem setup for muscle sample contraction, and the results of the convergence test for the numerical method are specified. (PDF) [file pone.0255027.s001.pdf]

S1 File

Additional details of the model and numerical method

The rule of the choice of the solution of Eq. (19) for Ca^{2+} concentration in subspace

Using an asymptotic approach at $\alpha_{SS} \to 0$ and Tikhonov theorem [47] we reduced the problem of finding the Ca^{2+} concentration is subspace c_{SS} to a solution of the cubic Eq. (19) at given values of R, c c_{SR}, and I_{CaL}. Depending of these values and the model parameters the equation can have one, two, or three real positive solutions. Fig. SM1 shows the plot of the left hand side of Eq. (19) at constant c = 0.5 μM, c_{SR} = 0.73 mM, I_{CaL} = 35 μM/s or 0 μM/s and different values of R.

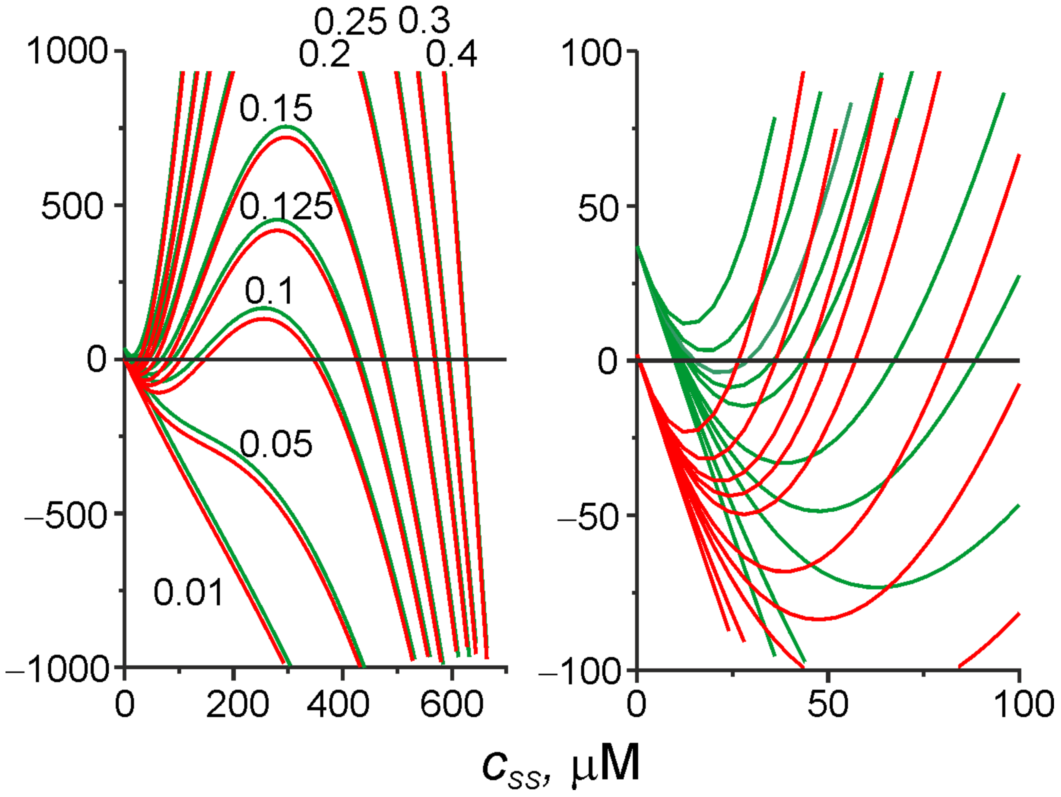

S1 Fig. 1. The plot of the left-hand side of Eq. (19). The left hand side value of Eq. (19) for different R values shown next to the curves and constant c = 0.5 μM, c_{SR} = 0.73 mM. The red lines correspond to I_{CaL} = 0 μM/s, the green lines correspond to I_{CaL} = 35 μM/s. *A* and *B* show the same plots at different scales.

For low or high R values, there is only one solution of the Eq. (19), for intermediate R values, there are three solutions. In special boundary cases, Eq. (19) can have one single and another repeated root.

S1 Fig. 2 shows the roots of Eq. (19) in the c_{ss}-R plane at constant c, c_{SR}, and positive I_{CaL}. An analysis of Eq. (17) shows that in the three-root case, the lowest and the highest ones are stable, while the intermediate one is unstable. As the Tikhonov theorem requires using only a stable root, we have to choose one of two.

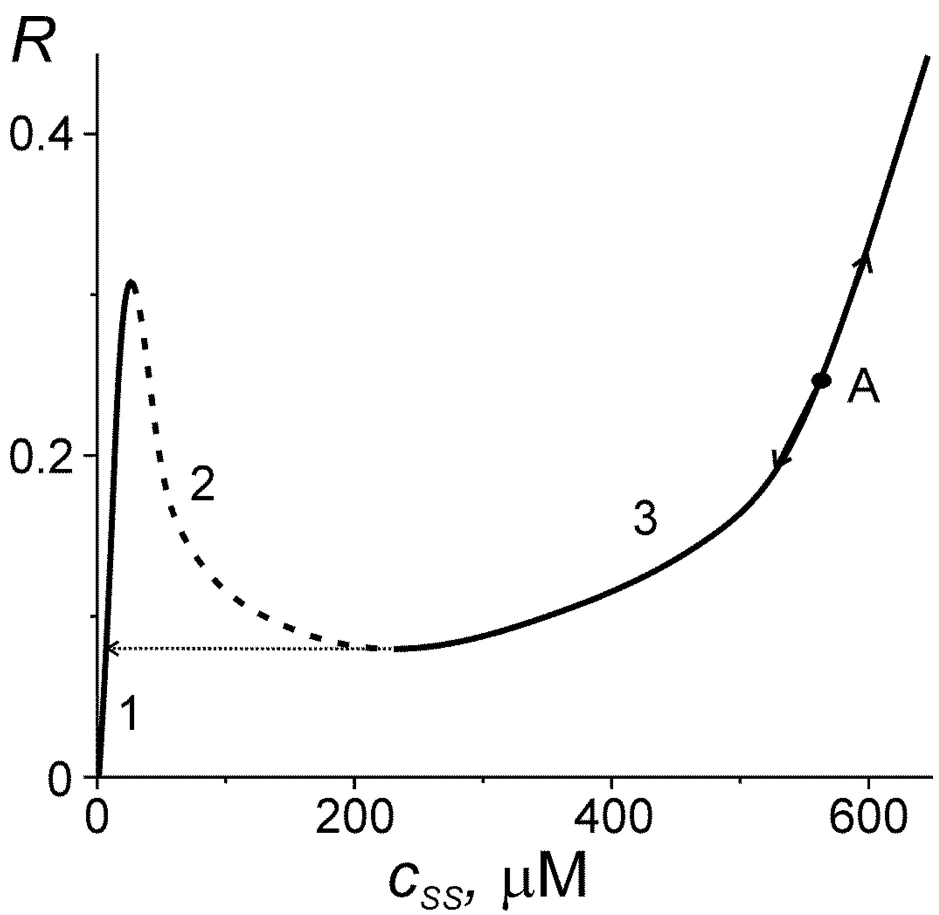

S1 Fig. 2. Roots of the Eq. (19) on the R–c_{Ss} plane at constant c, c_{SR}, and I_{CaL} = 35 μM/s. Solid lines 1 and 3 correspond to stable root branches for Eq. (17), the dashed line shows an unstable root branch 2.

When the slowly changing variables c, c_{SR}, and I_{CaL} vary, the plot shown in S1 Fig. 2 also changes while its topology remains the same. The left and the right ascending branches 1 and 3 are stable. Therefore, during numerical simulation, we were choosing a root on the same branch 1 or 3 as at the previous time step unless we reached one of two extremum points. These extremum points correspond to the transitions from the three-solution case to a one-solution case. When we reached an extremum during numerical integration, we jumped to another stable branch 1 or 3 and remained on it until the next bifurcation occurred. For example, if we were in point *A* on branch 3 (S1 Fig. 2) and move upwards (towards higher *R*), we remained on the same branch even after the transition caused by an increase in *R* above the transition point that corresponds to the local maximum on the left side of the plot. On contrary, if we start from the same point *A* and move downwards, a decrease in *R* below the second transition point (local minimum), we cannot remain on branch 3 and have to jump to branch 1. During further evolution of the system after the jump, we again remained on the same branch until it disappeared. As the initial conditions for a twitch simulation were taken at u = 0 (red lines in S1 Fig. 1), we started the simulation on the branch 1 in S1 Fig. 2. Jumps between branches 1 and 3 cause discontinuity of the solution c_{SS} seen in Fig. 2A corresponding to the instantaneous opening and closure of the CICR channels.

The 0D problem setup

In our model, myocardium is treated as transversally-isotropic material with hyperelastic passive stress and active stress caused by the actin-myosin interaction in cardiac muscle cells. The stress in myocardium is described by the constitutive equation

$$\mathbf{T} = \mathbf{T_{is}} - q\mathbf{E} + \left(\frac{T_a + T_{tit}}{l_s/l_{s0}}\right)\mathbf{B_f}, \qquad \mathbf{B_f} = \mathbf{f} \otimes \mathbf{f},$$

$$T_{is}^{ij} = \frac{\partial W_{el}}{\partial \varepsilon_{ij}}, W_{el} = c_{is1} e^{Q(I_1, I_2)},$$

$$Q = c_{is2}\left(0.25(I_1 - 3)^2 - 0.5(I_2 - 2I_1 + 3)\right).$$

Here **T** is the Cauchy stress tensor, $\mathbf{T_{is}}$ is its isotropic hyperelastic component specified by strain-energy exponential function W_{el} depending on the first and the second invariants of the right Cauchy-Green strain tensor or the Finger strain-tensor; q is pressure caused by myocardium incompressibility, **E** is a unit tensor, and the last term is an anisotropic stress caused by forces applied along muscle fibers to the area perpendicular to the fiber direction. The tensor $\mathbf{B_f}$ is a dyadic product of the strained unit vectors aligned with muscle fibers **f**; and scalars F_{act} and F_{tit} are an active tension generated by muscle and a passive tension of titin, a sarcomere protein connecting myosin filaments to Z-discs. T_a was defined by our model of myocardium mechanics (Eq. (14), [25]). The titin tension T_{tit} was described by an equation of worm-like chain model as described [25]; l_s and l_{s0} are the deformed and unstrained sarcomere lengths.

In the simulation of uniform uniaxial contraction of a cylindrical myocardial sample with strain $\lambda = {}^{l_s}/_{l_{s0}}$, the strained basis vectors **R** in cylindrical curvilinear coordinates are expressed through unit basis vectors **e** as

$$\begin{cases} \mathbf{R_r} = \dfrac{1}{\sqrt{\lambda}}\mathbf{e_r}, \\ \mathbf{R_\varphi} = \dfrac{1}{\sqrt{\lambda}}\mathbf{e_\varphi}, \\ \mathbf{R_z} = \lambda\mathbf{e_z}. \end{cases}$$

The equilibrium equations and the boundary conditions have the following form

$$\begin{cases} \boldsymbol{\nabla} \circ \mathbf{T} = 0, \\ u_z|_{z=0} = 0, \\ u_z|_{z=Ls} = 0 \; or \quad {}^{zz}|_{z=Ls} = F_e, \\ \mathbf{n} \circ \mathbf{T}|_{lateral\, surface} = 0, \end{cases}$$

where u_z are axial displacements, and n is an outward normal vector to free lateral surface of the muscle bundle. The constitutive equation then expressed through the Finger strain tensor $\mathbf{B} = \boldsymbol{\nabla}^{\mathbf{0}}\mathbf{R}^{\mathbf{T}} \cdot \boldsymbol{\nabla}\mathbf{R}$ as follows

$$\mathbf{T} = 2\left(\left(I_1(\mathbf{B})\frac{\partial W_{el}}{\partial I_2(\mathbf{B})} + \frac{\partial W_{el}}{\partial I_1(\mathbf{B})}\right)\cdot\mathbf{B} - \frac{\partial W_{el}}{\partial I_2(\mathbf{B})}\mathbf{B}^2\right) - p\mathbf{E} + (T_a + T_{tit})\lambda\mathbf{e_z}\mathbf{e_z}.$$

The components of the Finger tensor in unit unstrained reference coordinates are

$$B_{ij} = \begin{pmatrix} {}^1/_\lambda & 0 & 0 \\ 0 & {}^1/_\lambda & 0 \\ 0 & 0 & \lambda^2 \end{pmatrix}.$$

Thus, the first and second invariant of the Finger strain tensor are expressed as follows

$$I_1 = \lambda^2 + {}^2/_\lambda, I_2 = 2\lambda + {}^1/_{\lambda^2}.$$

One can express the Lagrange multiplier q from the boundary condition at lateral surface $T^{\varphi\varphi}$=0 and write down the following equation for full measurable muscle tensile tension T^{zz}

$$T^{zz} = T_a(n,\delta,\lambda) + T_{tit}(\lambda) + c_{is1}c_{is2}e^{Q(I_1,I_2)}\left(\lambda^3 - \lambda + {}^1/_{\lambda^2} - {}^1/_{\lambda^3}\right).$$

Convergence test for numerical method

A convergence test for the explicit Euler method for the set of ODEs of our model was performed by setting the time-step of 0.2, 0.1, and 0.05 ms (S1 Fig. 3).

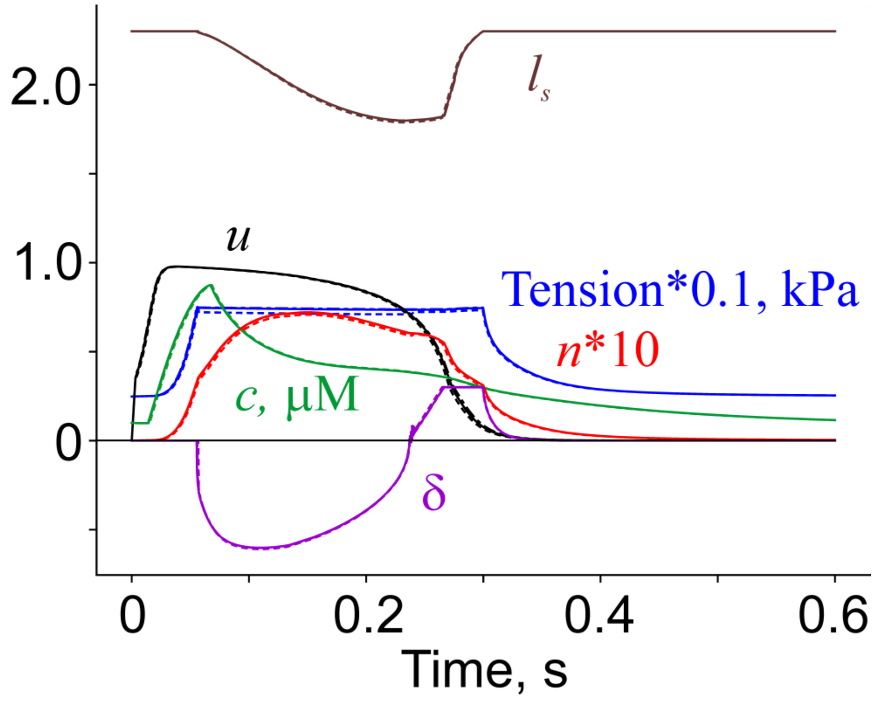

S1 Fig. 3. The results of the convergence analysis. Sarcomere length l_s (μm), dimensionless membrane potential u, normalized cross-bridge distortion δ, intracellular Ca^{2+} concentration c (μM), the fraction of actin-bound myosin heads n (multiplied by 10), and tension T (kPa,

multiplied by 0.1) are shown for time-steps 0.2 ms (dashed lines), 0.1 ms (continuous lines) and 0.05 ms (dot-dash lines).

A twitch isometric-isotonic contraction under a load of 7.5 kPa at an initial sarcomere length of 2.3 μm was simulated. At the time-step of 0.2 ms, there were numerical oscillations of δ when its value was slightly above zero; the time courses of membrane potential, the fraction of actin-bound myosin heads, tension, and sarcomere length were also slightly different from those obtained at smaller time-steps (S1 Fig. 3). Further decrease in the time-step from 0.1 ms to 0.05 ms did not cause any visible changes in the time-courses of these model variables. Therefore, the time step of 0.1 ms provides a good convergence of the numerical method.
