# Supplementary material for: Computationally efficient model of myocardial electromechanics for multiscale simulations
Source: PLoS One. 2021 Jul 22;16(7):e0255027. doi: 10.1371/journal.pone.0255027 (PMC8297763; doi:10.1371/journal.pone.0255027)
Supplement: Attachment — Submitted filename: PONE_response_2reviewers_Final.docx [file pone.0255027.s002.docx]

Dear prof. Panfilov,

We are very grateful to you and both reviewers for careful reading and evaluation of our manuscript and very useful comments and suggestions. We have prepared revised version of the manuscript with changes and additions suggested by the reviewers. The point to point responses to the reviewers’ comments are listed below.

Review Comments to the Author

Reviewer #1:

*Of course, very simplified description of the action potential generation used in the model does not imply its validation, since such action potential cannot be attributed not only to the human myocardium, but to any other species as well. This does not seem to be a drawback of the work, but rather its specific feature.*

*Nevertheless, this circumstance would be right to point out in the text somewhere among the limitations of the model.*

We agree with this comment and added a sentence concerning the action potential modelling and the validation of the electrophysiological block of the model.

MINOR COMMENTS

1. *Either at the end of the model description section or at the beginning of the section "Results", it would be helpful to state more clearly the 0D-conditions applied to the twitch simulations you ran in the 3D model. In particular, it should be clarified that for the correct simulation of the single cardiomyocyte behavior, you eliminated the factors of electrical and mechanical interaction between the cells in the 3D media, which was done by the identity of the cells' parameters and by their simultaneous stimulation.*

A sentence explaining the details of the simulations is added to the revised manuscript.

2. *The sentence «Following [19] we assumed that the cell membrane capacitance C normalized for its value at =1 and d1 depend on the axial strain ... as follows: .... »*

*on page 9 contains term d1 that previously arose only once within formula (2) on page 5 and was defined there in quite indirect way. This is very inconvenient for the readers. I'd suggest to clarify right after this phrase on page 9 that d1 is the longitudinal component of the conductivity tensor D.*

Additional explanations were added to the revised manuscript as suggested.

3. *It seems to be reasonable to add informative notations before the formal symbols A1 and A2 in section "Results" and in the respective figure legends. For example, such notations as «concentration of the calcium-troponin complexes in the overlap zone (A1)», «… in the non-overlap zone (A2)» would help readers to understand your results easier.*

The physical meaning of the variables A1 and A2 was added to the revised manuscript as suggested.

4. *Fig. 2 legend: «The time courses of model variables ... Ta (normalized for its maximal value)». It makes sense to explain here more precisely what maximum is meant. Probably you mean the absolute maximal value calculated for the steady-state conditions at saturating calcium concentration. However this is not explained. Meanwhile, by default the quoted phrase is perceived as normalizing to the force amplitude just of the presented twitch. Thus it becomes unclear why this amplitude in the figure turned out not equal to 1.*

The details for the tension normalizations are added to the Figure 2 legend in the revised manuscript.

5. *The conditions of the numerical experiment presented in Fig. 5B, and their differences from the experimental conditions corresponding to Fig. 5A are described very unclear. It should be clarified that the regular pacing with the rate of 1 Hz was stopped followed by one extrasystolic potentiation (for points corresponding to the intervals shorter than 1 s on panel 5B) or by one post-pause potentiation (for points corresponding to the intervals longer than 1 s on this panel).*

Explanations of the conditions of the numerical experiments shown in Fig. 5 were added to the revised manuscript as suggested.

Reviewer #2:

1. *It may be more appropriate to use the adjective "efficient" than "effective" in the title.*

The title of the revised manuscript was changes as suggested.

2. *The authors are strongly recommended to the clearly highlight the novel aspects of the proposed approach compared both to their own model (Syomin and Tsaturyan (2017) and to the similar models suggested in literature.*

A new subsection summarizing the novelty of our approach and results was added into the discussion section.

3. *In the numerical setting, the authors claim that the explicit Euler method gives convergent results for the time step dt=0.1 ms. This should be shown in a set of plots generated by convergence analyses reporting transients of some of the model outcomes obtained for different dt values.*

We are sorry for the mistyping in the first para of Results section that could be misleading: the time-steps tested for the convergence analysis were 0.2, 0.1, and 0.05 ms. The results of the convergence analysis are provided in S1 Fig. 3 of the Supporting information file of the revised manuscript.

4. *Although the governing equation of electrophysiology (1) is a transient PDE, in the numerical analyses, the authors seem to solve solely a set of ODEs. Therefore, it is unclear whether the claims/findings concerning the reduction of stiffness and convergence with respect to the time step hold also for PDEs including the conservation of linear momentum.*

In the manuscript revised, we indeed solved solely a system of ODEs. However, we also tested the model for the simulation of 2D processes. In this case, we implemented the finite element method for finding transmembrane potential and mechanical displacements in the nodal points and numerically solved ODEs for all other kinetic variables using the explicit Euler method. The time-step of 0.1 ms provided good convergence, as it did for the solution of the ODE system for the 0D simulations presented in the manuscript and Supporting Information. The details of the 2D simulations will be published elsewhere.

5. *In the manuscript, some abbreviations such as TnC, Tpm-Tn remain undefined. The meaning of all the abbreviations mush be explained.*

All abbreviations are introduced in the revised manuscript as suggested.

Yours, sincerely

Fyodor Syomin, PhD

Research Scientist

Department of Biomechanics

Institute of Mechanics

Lomonosov Moscow State University
